# Who perishes more? Trait-mediated mortality of vertebrates during installation of wind and solar energy infrastructure in a neotropical dry forest

**DOI:** 10.1007/s00267-026-02661-4

**Published:** 2026-11-14

**Authors:** Elisson Ygours Costa de Lima, Raul Fernandes Dantas Sales, Eliza Maria Xavier Freire

**Affiliations:** 1 Programa Regional de Pós-graduação em Desenvolvimento e Meio Ambiente, Universidade Federal do Rio Grande do Norte, Natal, Brasil; 2 Departamento de Botânica e Zoologia, Laboratório de Herpetologia, Universidade Federal do Rio Grande do Norte, Centro de Biociências, Natal, Brasil

**Keywords:** Renewable energy, Habitat, Body form, Herpetofauna, Caatinga.

## Abstract

The transition to renewable energy has been widely promoted as a response to the environmental impacts of fossil fuel–based systems intensified since the Industrial Revolution. However, renewable energy infrastructure can also generate localized ecological effects on wildlife. From a functional perspective, it is therefore critical to examine how traits of vertebrates shape vulnerability to mortality under environmental disturbance associated with the installation phase of wind and solar developments, particularly during vegetation clearing and earthmoving. We investigated mortality associated with the installation of five renewable energy facilities (four wind farms and one solar plant) affecting wild vertebrates in the Caatinga of Rio Grande do Norte State, northeastern Brazil. Faunal rescue and deterrence programs conducted during installation recorded 29,099 individuals between 2020 and 2025. Live animals were relocated to designated release areas, whereas dead individuals were deposited in zoological collections. Species-level mortality proportions were used as comparative metrics. A beta regression model revealed that reptiles exhibited significantly higher mortality than amphibians, mammals and birds. A second beta regression model including habitat and body form simultaneously indicated significant associations of both predictors with mortality. In the joint model, estimated mortality was significantly lower for arboreal species than for fossorial and terrestrial species. Snake-like species exhibited the highest estimated mortality, significantly exceeding quadrupeds and winged species. These results demonstrate non-random mortality strongly associated with functional traits that limit locomotor performance and behavioral avoidance, highlighting the need to incorporate trait-based vulnerability into impact assessment and mitigation protocols during early infrastructure installation in biologically sensitive ecosystems.

## Introduction

Since prehistory, the use of energy has been essential to human development, with the control of fire representing an early milestone in harnessing natural resources to meet basic needs (Smil 2017). Subsequently, the Neolithic Revolution consolidated the use of biomass, primarily fuelwood, as the main energy source (Sieferle 2001), until the Industrial Revolution introduced a paradigmatic shift with the incorporation of fossil fuels such as coal, oil, and natural gas to sustain mechanization and economic expansion (Wrigley 2010; Malm 2016).

This productivist model resulted in a rupture in the relationships between society and nature, contributing to resource depletion and ecosystem degradation, with direct interference in climate change (Foster 2000; Porto-Gonçalves 2004). In this context, the energy transition has emerged as a strategic and urgent response to climate challenges, promoting the gradual replacement of fossil fuels with clean and renewable alternatives such as solar, wind, hydropower, and modern biomass (Olabi and Abdelkareem 2022), particularly within the international agreements established by the United Nations 2030 Agenda (United Nations 2015).

Among these alternatives, wind and solar energy stand out within the scope of so-called “clean energy”, as they present substantially reduced environmental impacts compared with fossil-based systems, especially due to the absence of greenhouse gas (GHG) emissions during operation, thereby contributing to climate change mitigation and the promotion of a more sustainable energy model (IPCC 2023). In Brazil, the energy transition has advanced significantly, driven by public policies and the adoption of clean technologies. Renewable sources represent 85% of the national electricity matrix, with hydropower accounting for 50%, wind power for 16%, solar power for 10%, and biomass for 8%, consolidating the country as one of the leading renewable energy producers in Latin America (ANEEL 2026).

However, despite their benefits in reducing atmospheric emissions, wind and solar energy sources have generated controversy within the discourse of clean energy due to their adverse environmental impacts. In northeastern Brazil, studies indicate that wind farms have produced negative effects across social and environmental contexts (Gorayeb et al. 2018; Hofstaetter and Pessoa 2022). During operation, these developments affect volant fauna through fatal bird collisions with turbines and panels and barotrauma in bats (Baerwald et al. 2008; Bennun et al. 2021; Flemming 2025). Both volant and non-volant fauna are also impacted through habitat loss, noise and light pollution, electrocution, altered ecological dynamics, and intensified hunting, often exacerbated by the absence of effective monitoring (Bennun et al. 2021; Flemming 2025).

Although the impacts of the operational phase of wind and solar developments are well documented, the effects of the installation phase have received less attention. During wind farm installation, activities such as vegetation clearing, earthmoving, and road construction cause significant alterations to the natural environment and may result in animal mortality due to vehicle strikes and burial (Freire et al. 2023). In solar power plants, these actions occur on an even larger scale due to the need for extensive flat and open areas, intensifying native vegetation removal and compromising soil permeability, thereby aggravating erosive processes and destroying natural shelters used by various species (Barbosa-Filho et al. 2015). In addition to direct mortality, the use of heavy machinery and the acoustic disturbances generated during installation cause behavioral disruptions that may lead animals to flee to adjacent areas, increasing the risk of new conflicts with humans (Treves 2009; Zarzavilla et al. 2022).

Given this context, it is essential to examine how both taxonomic affiliation and functional traits shape vertebrate vulnerability to mortality during the installation phase of wind and solar developments, particularly during vegetation clearing and earthmoving. Amphibians and reptiles show strong dependence on specific microhabitats and have morphological constraints limiting dispersal capacity (Vanhooydonck et al. 2002; Pianka and Vitt 2003), factors that may make them more susceptible to these impacts. This susceptibility might be associated with a predominance of body forms that would show more vulnerability to vegetation and soil disturbances, specially elongated bodies and absence of limbs. Elongated body forms are widely interpreted as adaptations facilitating locomotion through soil and other confined substrates (Shine and Wall 2008; Morinaga and Bergmann 2020; Anelli et al. 2024). Consequently, disturbance caused by vegetation clearing and earthmoving may disproportionately expose these species to direct mortality.

Accordingly, this study addresses the following question: Which biological attributes are associated with species susceptibility to mortality during the installation of wind and solar infrastructure? Specifically, we evaluate whether mortality differs among major vertebrate groups (amphibians, reptiles, birds, and mammals) and whether species’ primary habitat and body form explain additional variation in mortality risk. We hypothesize that mortality during the installation phase is primarily determined by species’ functional traits, with ecological and morphological characteristics influencing their ability to avoid habitat disturbance. Based on this hypothesis, we predict that reptiles will exhibit higher mortality than other vertebrate groups because they disproportionately comprise species with ecological and morphological traits associated with reduced escape capacity during vegetation clearing and soil disturbance. We further predict that fossorial species and species with elongated, snake-like body forms will experience the highest mortality due to their reduced escape performance and greater exposure to soil disturbance during vegetation clearing and earthmoving.

## Methods

### Study Object

**Fig. 1:**
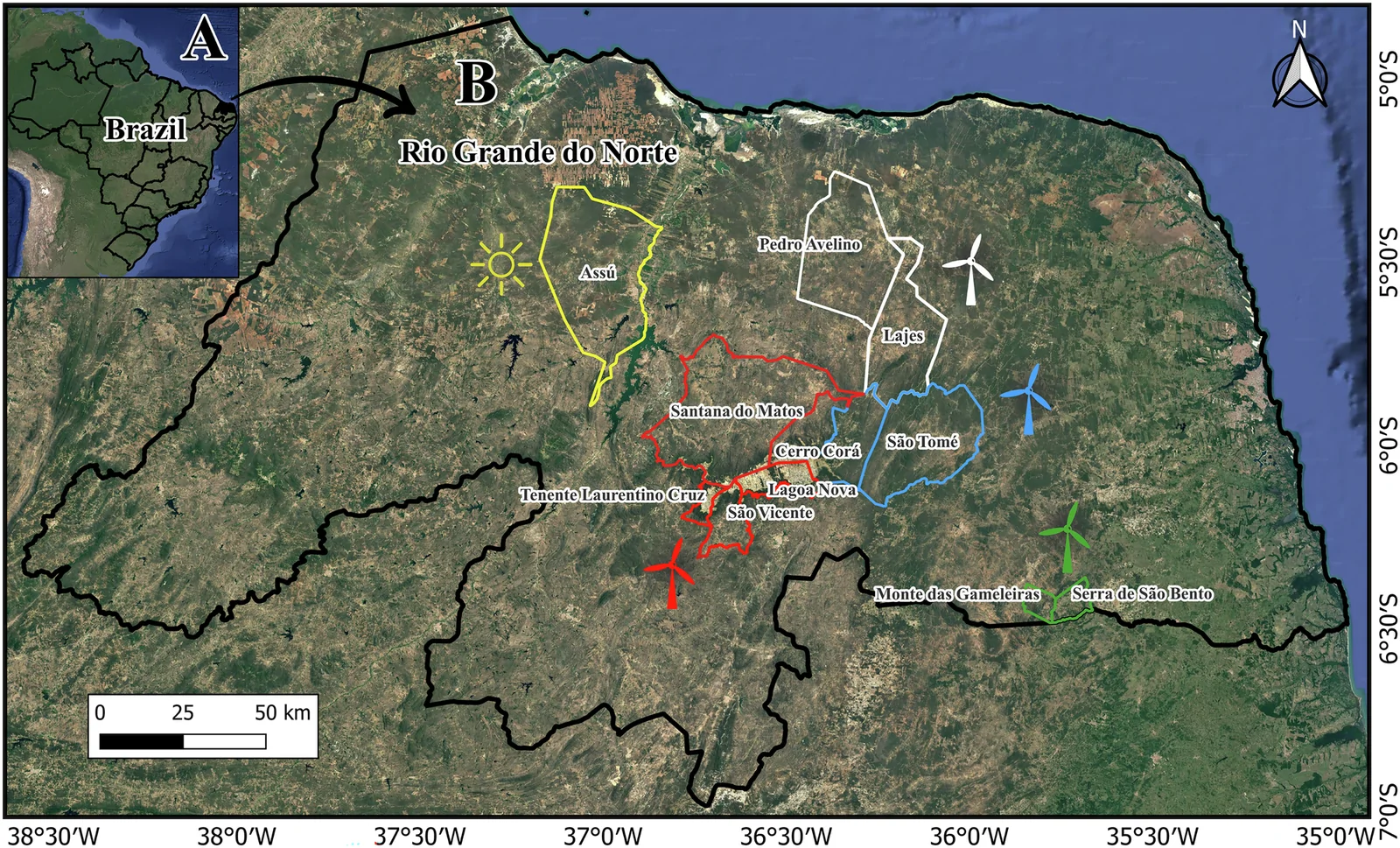
Location of Rio Grande do Norte (RN) in Brazil (A), showing (B) the municipalities where the five renewable energy developments analyzed in this study are located

Data were obtained from faunal rescue and deterrence programs conducted during the installation phase of five renewable energy developments (four wind farms and one photovoltaic solar plant) in Rio Grande do Norte State (RN), northeastern Brazil (Fig. 1), between 2020 and 2025. About 95% of the RN territory is within the semiarid Caatinga region, which is the largest and most continuous expanse of seasonally dry tropical forest biome in the world (Queiroz et al. 2017). RN is a leading state in wind and solar power generation due to favorable environmental conditions of wind availability and solar irradiance. The developments are located in rural areas of the following municipalities: Development 1 (wind energy; installation from March 2021 to April 2022)—São Vicente, Santana do Matos, Tenente Laurentino Cruz, and Lagoa Nova; Development 2 (wind energy; installation from February 2020 to January 2021)—Cerro Corá, Lajes, and São Tomé; Development 3 (wind energy; installation from May 2021 to March 2023)—Lajes and Pedro Avelino; Development 4 (wind energy; installation from September 2022 to April 2023)—Serra de São Bento, Monte das Gameleiras, and São José do Campestre; Development 5 (photovoltaic solar energy; installation from June 2023 to February 2025)—Assú (Fig. 1). The data of faunal programs were provided by environmental consulting companies responsible for implementing the programs, together with voucher specimens deposited in the zoological collections of the Federal University of Rio Grande do Norte (UFRN).

Faunal rescue and deterrence programs are conducted by biologists and veterinarians during the construction (installation) phase of the developments, specifically in activities involving vegetation clearing, earthmoving, and blasting for the opening of access roads and construction of turbine platforms or solar panel installation areas, as well as other associated facilities such as substations and transmission lines. Professionals accompany the construction activities, deterring and rescuing animals from affected areas and subsequently relocating them to predefined release sites. Animals that are passively deterred due to visual or auditory disturbances caused by the activity itself are also recorded. Animals injured by machinery receive veterinary care and are monitored prior to release, whereas animals that die (see some examples in Online Resource 1) are collected and deposited in scientific collections. The implementation of faunal rescue and deterrence programs, as well as other environmental programs, is a requirement of installation licenses issued by RN state environmental regulatory agency (IDEMA-RN).

### Statistical Analysis

For this study, raw data spreadsheets from each development were initially evaluated for data provenance. Records were categorized into five broad faunal groups: Arthropoda (arthropods), Amphibia (amphibians), Reptilia (non-avian reptiles), Aves (birds), and Mammalia (mammals). Records with doubtful identification (e.g., species not occurring in RN), lacking taxonomic identification, or identified only to the genus level or higher were excluded. Additionally, records of isolated bird nests and insect colonies (e.g., bee hives) were also excluded. Species-level mortality analyses were conducted only for the vertebrate groups due to the low reliability of taxonomic identification for arthropods.

Each recorded species was categorized according to its primary habitat into four categories: fossorial, terrestrial, arboreal, and aquatic, based on the literature (Sick, 1997; Reis et al. 2006; Mesquita et al. 2017; França et al. 2020). For analytical simplicity, species described in the literature as semi-arboreal were treated as arboreal, and those described as semifossorial were treated as fossorial. Aquatic species were excluded from habitat analyses because the installation of wind and photovoltaic facilities primarily affects terrestrial environments through vegetation clearing and soil disturbance. Therefore, habitat-related hypotheses were intended to compare species directly exposed to these terrestrial disturbances. The low number of aquatic species (*n* = 2) further precluded meaningful statistical comparisons.

Each species was also classified according to its predominant body form into five categories: snake-like, terrestrial quadruped, scansorial quadruped, burrowing quadruped, and winged. These categories were defined based on the locomotor morphology and overall body plan of each species. Species with elongated bodies and reduced or absent limbs were classified as snake-like; volant species (birds and bats) as winged; terrestrial mammals, lizards and amphibians with cursorial habits as terrestrial quadrupeds; climbing mammals, lizards and amphibians as scansorial quadrupeds; and fossorial mammals and amphibians with robust digging adaptations as burrowing quadrupeds. Body-form categories were assigned based on species morphology and locomotor habits described in the literature (Vitt and Caldwell 2014; Pough et al. 2022).

The response variable analyzed in this study was species-level mortality proportion, with species considered the unit of inference. Consequently, individuals that were rescued alive, including those requiring veterinary treatment, were classified as survivors rather than mortality events. Although these individuals were affected by installation activities, injury severity was not consistently recorded in the original datasets, precluding standardized analyses of sublethal impacts. Therefore, the present study focuses exclusively on mortality as a robust and consistently documented endpoint across all developments.

Because the objective was to identify biological attributes associated with species vulnerability, each species contributed a single mortality estimate to the analyses, regardless of the total number of individuals recorded. Because species differed markedly in the number of recorded individuals, mortality was expressed as a species-level proportion (number of deaths divided by the total number of records), thereby standardizing mortality estimates among species independently of sample size. Species with few records were excluded from the analyses to reduce the influence of unreliable mortality estimates derived from very small sample sizes. Given the generally lower encounter rates and more elusive behavior of mammals in fauna rescue and monitoring, the minimum inclusion threshold for this group was set at five records, whereas a threshold of ten records was applied to amphibians, reptiles, and birds.

Mortality proportions were analyzed using generalized linear modeling approaches, which provide an appropriate framework for modeling non-normal response variables, including proportions (McCullagh & Nelder, 1989; Zuur et al. 2009). Specifically, beta regression models with a logit link function were fitted because they are specifically designed for continuous response variables bounded between 0 and 1 and are therefore well suited for modeling proportional ecological data (Ferrari & Cribari-Neto, 2004). Because beta regression requires response values to lie within the open interval (0,1), mortality proportions were transformed following the approach proposed by Smithson and Verkuilen (2006), which accommodates observations equal to zero while preserving the relative differences among species. This transformation was necessary because 54 of the 153 analyzed species exhibited zero mortality, whereas no species exhibited complete (100%) mortality.

To determine whether mortality differed among taxonomic groups, faunal group (Amphibia, Reptilia, Mammalia, and Aves) was included as the explanatory variable in a beta regression model. To evaluate whether ecological traits influenced mortality during the installation of renewable energy facilities, a second beta regression model included species’ primary habitat (arboreal, terrestrial, and fossorial) and body form (snake-like, terrestrial quadruped, scansorial quadruped, burrowing quadruped, and winged) as explanatory variables. Habitat and body form were included as additive predictors in the same beta regression model, with no interaction term.

The significance of explanatory variables was assessed using likelihood-ratio tests. When significant effects were detected, pairwise comparisons among estimated marginal means were performed using Tukey-adjusted P values. Multicollinearity between habitat and body form was evaluated using generalized variance inflation factors (GVIF), and no evidence of problematic collinearity was detected (all GVIF^(1/(2×df)) < 2). As a sensitivity analysis, alternative model structures, including beta regression with habitat-dependent precision and quasibinomial generalized linear models, were also evaluated, yielding qualitatively identical biological conclusions. All analyses were performed in R version 4.6.1 using the packages betareg (Cribari-Neto and Zeileis 2010), car (Fox and Weisberg 2019), and emmeans (Lenth and Piaskowski 2026).

## Results

### Faunal Groups

**Fig. 2:**
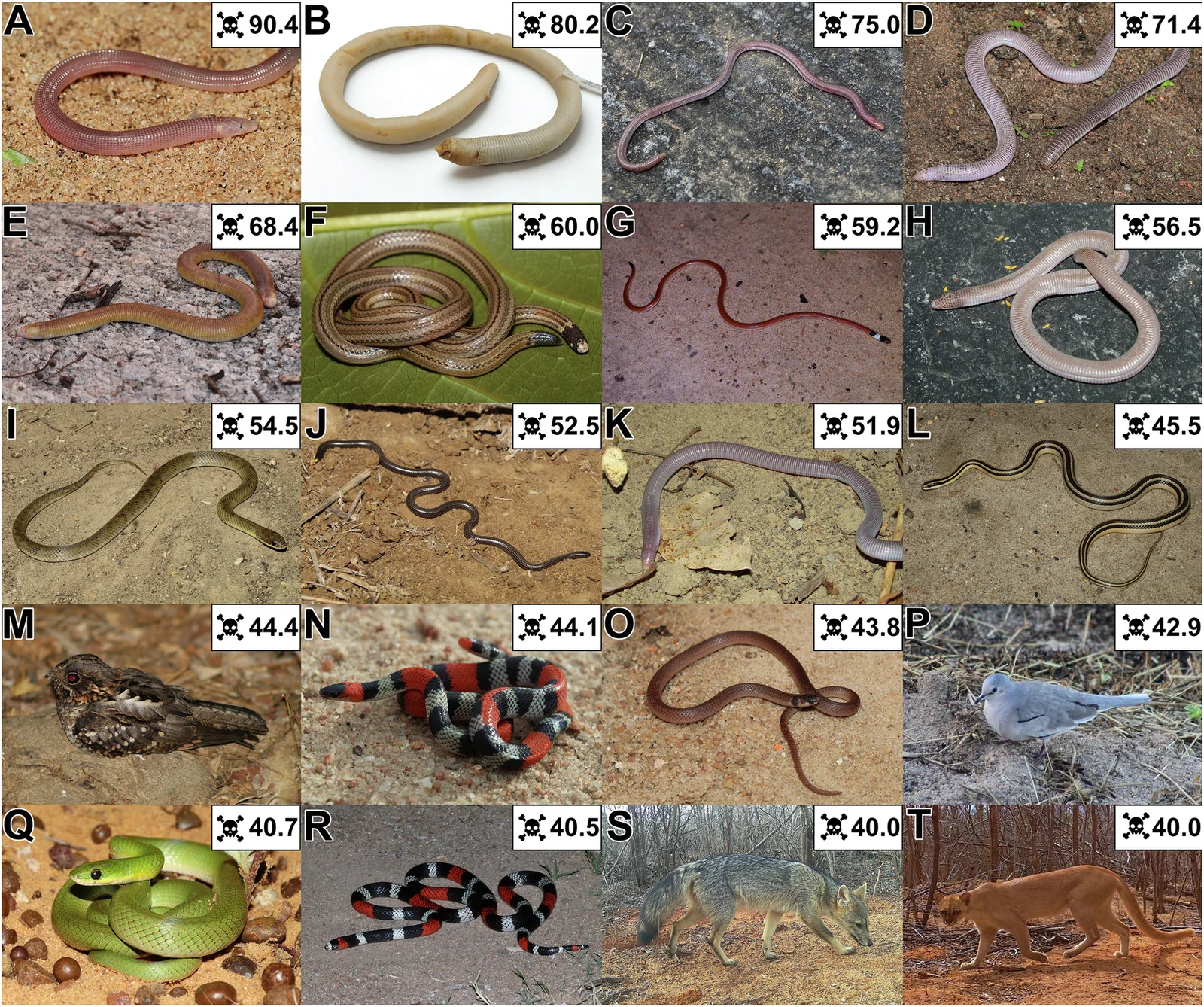
The 20 vertebrate species with the highest mortality proportions recorded by faunal rescue and deterrence programs conducted during the installation of five wind and photovoltaic energy developments in Rio Grande do Norte State, northeastern Brazil. **A** *Amphisbaena heathi*; **B** *Leposternon polystegum*; **C** *Amphisbaena carvalhoi*; **D** *Amphisbaena littoralis*; **E** *Amphisbaena alba*; **F** *Apostolepis longicaudata*; **G** *Apostolepis cearensis*; **H** *Amphisbaena vermicularis*; **I** *Erythrolamprus poecilogyrus*; **J** *Epictia borapeliotes*; **K** *Amphisbaena pretrei*; **L** *Lygophis dilepis*; **M** *Hydropsalis parvula*; **N** *Oxyrhopus trigeminus*; **O** *Tantilla melanocephala*; **P** *Columbina picui*; **Q** *Erythrolamprus viridis*; **R** *Micrurus bonita*; **S** *Cerdocyon thous*; **T** *Herpailurus yagouaroundi*. The values in the white boxes in the upper right corner of each photo represent the mortality proportion (%). Photos: A–S: Raul Sales; T: Bruno França

The compilation of data from faunal rescue and deterrence programs conducted during the installation of the five analyzed developments yielded 29,099 faunal records, including 1,925 arthropods (6.6%), 1,295 amphibians (4.5%), 20,614 reptiles (70.8%), 4,302 birds (14.8%), and 963 mammals (3.3%). The records encompassed 215 vertebrate species, including 123 birds, 59 reptiles, 21 mammals, and 12 amphibians. Of this total, 153 species (77 birds, 49 reptiles, 15 mammals, and 12 amphibians) met the minimum sample size criteria for inclusion in the statistical analyses. Among the 20 vertebrate species with the highest mortality proportions, 16 species are limbless snake-like reptiles (nine snakes and seven amphisbaenians), along with two birds and two mammals (Fig. 2, Online Resource 2).

**Fig. 3:**
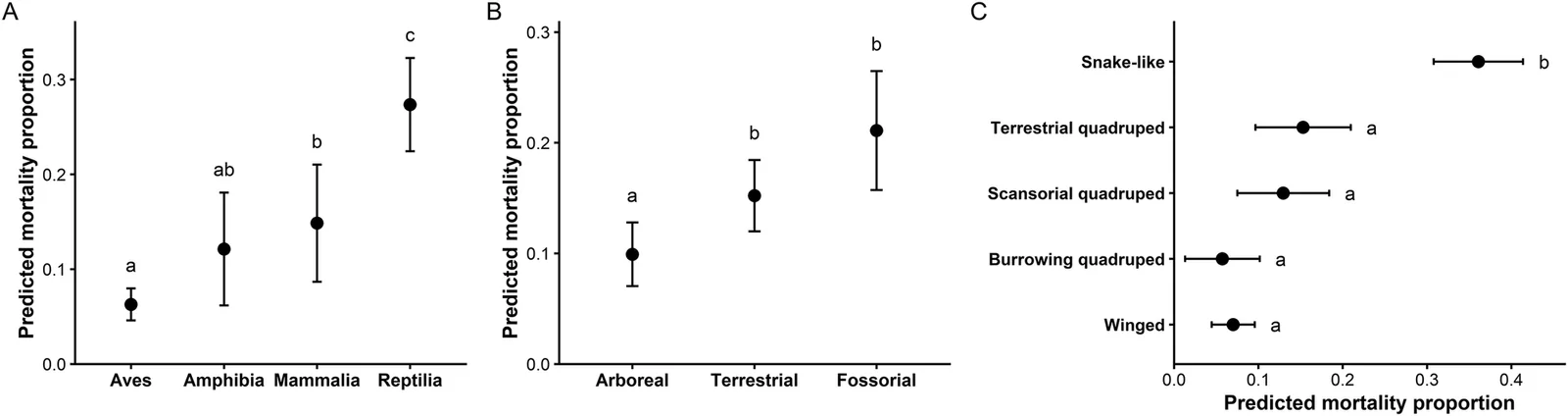
Estimated species-level mortality proportions (±95% confidence intervals) obtained from beta regression models for (**A**) faunal groups, **B** habitat categories, and **C** body forms. Values shown in (**B** and **C**) were obtained from the joint beta regression model including habitat and body form simultaneously. Mortality proportions represent the estimated proportion of dead individuals relative to the total number of recorded individuals for each species. Different letters above the estimates indicate statistically significant differences among categories based on Tukey-adjusted pairwise comparisons (*P* < 0.05)

Mortality proportions differed significantly among faunal groups (likelihood-ratio test, χ² = 92.69, df = 3, *P* < 0.001). Beta regression estimated the highest mortality for reptiles, followed by mammals, amphibians, and birds (Table 1, Fig. 3A). Pairwise comparisons (Table 2) revealed that reptiles exhibited significantly higher mortality than amphibians, mammals and birds. Mammals also showed significantly higher mortality than birds, whereas mortality did not differ significantly between amphibians and birds or between amphibians and mammals (Table 2).

**Table 1:** Estimated species-level mortality proportions obtained from beta regression models based on the pooled data from faunal rescue and deterrence programs conducted during the installation of five wind and photovoltaic energy developments in Rio Grande do Norte State, northeastern Brazil

Predictor	Estimated mortality (%)	95% CI
**Faunal groups**		
Birds	6.3	4.6–8.0
Amphibians	12.1	6.2–18.1
Mammals	14.9	8.7–21.0
Reptiles	27.4	22.4–32.3
**Habitat**		
Arboreal	9.9	7.0–12.8
Terrestrial	15.2	12.0–18.4
Fossorial	21.1	15.7–26.5
**Body form**		
Winged	7.0	4.5–9.6
Burrowing quadruped	5.7	1.3–10.2
Scansorial quadruped	12.9	7.5–18.4
Terrestrial quadruped	15.3	9.6–21.0
Snake-like	36.1	30.8–41.4

**Table 2:** Significant pairwise comparisons of estimated marginal means (estimated mortality proportions) obtained from beta regression models with Tukey-adjusted *P* values. Habitat and body-form comparisons were obtained from the joint additive model, in which both predictors were included simultaneously. Estimates represent differences in mortality proportions between groups

Comparison	Estimate	SE	z	P
**Faunal group**				
Amphibia x Reptilia	−0.152	0.038	−3.965	<0.001
Aves x Mammalia	−0.086	0.032	−2.723	0.033
Aves x Reptilia	−0.211	0.025	−8.304	<0.001
Mammalia x Reptilia	−0.125	0.039	−3.183	0.008
**Habitat (adjusted for body form)**				
Arboreal x Fossorial	−0.112	0.033	−3.350	0.002
Arboreal x Terrestrial	−0.053	0.021	−2.490	0.034
**Body form (adjusted for habitat)**				
Snake-like x Burrowing quadruped	0.3037	0.0334	9.089	<0.001
Snake-like x Scansorial quadruped	0.2315	0.0417	5.556	<0.001
Snake-like x Terrestrial quadruped	0.2079	0.0412	5.042	<0.001
Snake-like x Winged	0.2909	0.0324	8.986	<0.001

### Habitat and Body Form

A beta regression model including habitat and body form simultaneously indicated that both predictors were significantly associated with mortality (habitat: χ² = 13.88, df = 2, *P* < 0.001; body form: χ² = 57.44, df = 4, *P* < 0.001). The model showed good overall fit (pseudo-R² = 0.659). In the joint model, estimated mortality was significantly lower for arboreal species than for fossorial and terrestrial species, whereas fossorial and terrestrial species did not differ significantly (Table 2). Estimated mortality increased from arboreal to terrestrial and fossorial species (Table 1, Fig. 3B).

In the same joint model, snake-like species exhibited the highest estimated mortality (Table 1, Fig. 3C), significantly exceeding burrowing quadrupeds, scansorial quadrupeds, terrestrial quadrupeds, and winged species (Table 2). No other pairwise comparisons were significant.

## Discussion

The results of this study show that the installation phase of wind and solar energy developments exerts substantial mortality impacts on Caatinga fauna and that these impacts are not evenly distributed across vertebrate taxa but strongly concentrated in reptiles. Habitat disturbance associated with vegetation removal and soil modification required for infrastructure installation increase exposure to risk and elevate mortality for reptiles, a pattern likely associated with prevalent morphological characteristics in this group, such as elongated bodies, short or reduced limbs, and low body profile, which directly influence locomotor performance on open or compacted surfaces. As discussed by Vanhooydonck et al. (2002), these locomotor constraints reduce escape capacity and increase exposure to direct threats. Thus, the combination of morphology, low mobility, and dependence on specific microhabitats contributes to the disproportionate impact observed in reptiles.

On the other hand, amphibians did not constitute a representative group in terms of number of records, nor were they severely impacted in terms of mortality. Amphibians have life histories directly associated with water body habitats (Vieira et al. 2009). Moreover, the Caatinga region is characterized by low precipitation and prolonged drought periods, during which amphibians typically burrow primarily in the beds of temporary rivers, where they remain in estivation (Jared et al. 2020). Although this behavior, combined with locomotor constraints, may render amphibians vulnerable to soil disturbance, the relatively limited mortality observed for this vertebrate group is likely related to the fact that the installation of wind farms and solar facilities usually does not encompass water bodies, which are legally protected permanent preservation areas under the Brazilian Forest Code (Law No. 12,651/2012).

Birds and mammals also constituted vertebrate groups that experienced lower direct mortality than reptiles. Birds have winged body forms and benefit from flight, which enables rapid dispersal from disturbed areas and explains why most recorded species (96 of 123) were not affected in terms of mortality. Nevertheless, two bird species ranked among the most impacted by construction activities, likely due to species-specific behavioral traits. One species (*Columbina minuta*) is synanthropic, tolerant of human presence, and frequently forages on the ground (Fontoura and Orsi 2013), while the other species (*Hydropsalis torquata*) is nocturnal, terrestrial and nests on the ground (Almeida et al. 2022), characteristics that increase vulnerability to soil-disturbing activities.

For mammals, the capacity for flight in bats and the pronounced wariness and escape abilities of non-volant species, both on the ground and in climbing, help explain the lower direct mortality impacts observed in this group when compared to reptiles. However, the use of daytime shelters in tree cavities or underground burrows by non-volant mammals (Giordano 2015; Reis et al. 2006) confers some vulnerability to vegetation clearing and earthmoving. Accordingly, the destruction of shelter sites during periods of inactivity is the most plausible explanation for the high mortality proportions observed in *Cerdocyon thous* and *Herpailurus yagouaroundi*, as well as for recorded deaths in other mammal species.

Our findings suggest that species traits, rather than taxonomy itself, provide a more mechanistic explanation for mortality risk during infrastructure installation. Habitat use emerged as an important predictor of mortality, with fossorial and terrestrial vertebrates exhibiting consistently higher mortality than arboreal species. This pattern is consistent with the direct effects of vegetation removal and soil disturbance, which destroy underground refuges and expose animals that normally remain protected beneath the soil surface. Fossorial species may therefore experience mortality not only because they are directly injured during earthmoving activities but also because the destruction of subterranean microhabitats greatly reduces opportunities for escape. Although the raw mortality distributions suggested higher mortality among fossorial than terrestrial species (Online Resource 3), this difference was not significant when habitat and body form were included simultaneously in the model, indicating that the initial pattern was largely explained by differences in body form among habitat categories.

Beyond habitat use, body form independently explained variation in mortality among species. Snake-like species exhibited substantially higher mortality than all other body-form categories when habitat was included simultaneously in the joint model. The elongated limbless body typical of snakes and amphisbaenians facilitates movement through soil and other confined substrates (Morinaga and Bergmann 2020), but may reduce escape efficiency during rapid habitat disturbance, particularly under heavy machinery and soil excavation (Lovich and Ennen 2011). The most affected snake-like species were amphisbaenians, which exhibit highly specialized morphological and physiological adaptations for life in subterranean tunnels, including reinforced skulls, cylindrical bodies, absence of limbs, as well as behavioral strategies related to thermoregulation and microhabitat selection under specific thermal and moisture conditions (Gans 1978; Navega-Gonçalves and Benites 2019). Although advantageous for burrowing and subterranean persistence, these attributes entail strong dependence on specific microhabitats and low mobility, rendering these organisms particularly vulnerable to physical soil disturbances.

Technical studies assessing environmental impacts and supporting environmental licensing for wind and solar energy developments typically associate habitat loss and fragmentation with the installation phase, whereas faunal mortality is generally linked only to the operational phase, particularly in volant fauna (Bennun et al. 2021; Flemming 2025). In contrast, the present study demonstrates that the installation phase affects a different suite of species through fundamentally different ecological mechanisms. According to Lovich and Ennen (2011), the construction of solar and wind facilities in arid regions of USA has been shown to cause habitat fragmentation, soil compaction, and vegetation alteration, directly affecting low-mobility species dependent on specific microhabitats (Lovich and Ennen 2011). These authors emphasize that conventional mitigation protocols frequently fail to adequately account for fossorial or range-restricted fauna, contributing to the under-recognition of actual impacts on these groups. Furthermore, Northrup and Wittemyer (2013) argue that infrastructure installation can alter migratory routes and increase direct vertebrate mortality, particularly in areas characterized by sparse vegetation and high solar exposure.

From this perspective, our findings indicate that mortality of multiple animal species, especially limbless squamates with fossorial habits, during installation of renewable energy developments in northeastern Brazil represents a neglected environmental impact, revealing limitations in the effectiveness of prevention and control measures and contradicting principles established in Brazilian environmental legislation. Due to their subterranean habits and low detectability, these animals are often not adequately considered in conventional deterrence and rescue methodologies, leaving them vulnerable to vegetation removal and physical soil disturbance. This operational gap compromises the scope of environmental mitigation programs and conflicts with the precautionary principle established in environmental law by failing to ensure adequate protection for all potentially affected species. These impacts may be further exacerbated by recent changes to environmental licensing legislation in Brazil (Law No. 15,190/2025), which introduce new licensing modalities and relax prior assessment and monitoring requirements (Jorge and Caliman 2026).

## Conclusion

Overall, our findings demonstrate that mortality during the installation of renewable energy facilities is strongly trait-dependent. Rather than affecting vertebrate species uniformly, installation activities disproportionately impact species possessing combinations of ecological and morphological characteristics that limit their ability to avoid habitat disturbance. Our analyses focused exclusively on direct mortality, whereas other responses, including displacement, changes in habitat use, reduced reproductive success, and alterations in movement behaviour, were beyond the scope of this study. Future studies integrating both lethal and non-lethal responses will provide a more comprehensive understanding of how different vertebrate groups are affected during the installation of renewable energy infrastructure.

Although environmental licensing is supported by normative instruments, it still fails to incorporate methodologies capable of adequately detecting and mitigating faunal impacts. Our findings show that renewable energy installation can cause substantial direct mortality through changes in habitat structure, especially among fossorial, snake-like reptiles. These results therefore reinforce the need to revise environmental protocols, with particular attention to these species. Furthermore, infrastructure planning should incorporate prior data on local faunal abundance and distribution to avoid interventions in ecologically sensitive areas. Adoption of these strategies may substantially improve the effectiveness of management programs and help ensure that expansion of the energy matrix proceeds in a manner compatible with biodiversity conservation.

## Supplementary information

MS Lima et al_Supplementary material_revised

## Data Availability

Data will be made available on request.
