# Supplementary material for: Who perishes more? Trait-mediated mortality of vertebrates during installation of wind and solar energy infrastructure in a neotropical dry forest
Source: Environ Manage. 2026 Nov 14;76(11):351. doi: 10.1007/s00267-026-02661-4 (PMC13769722; doi:10.1007/s00267-026-02661-4)
Supplement: Supplementary file 1 [file 267_2026_2661_MOESM1_ESM.pdf]

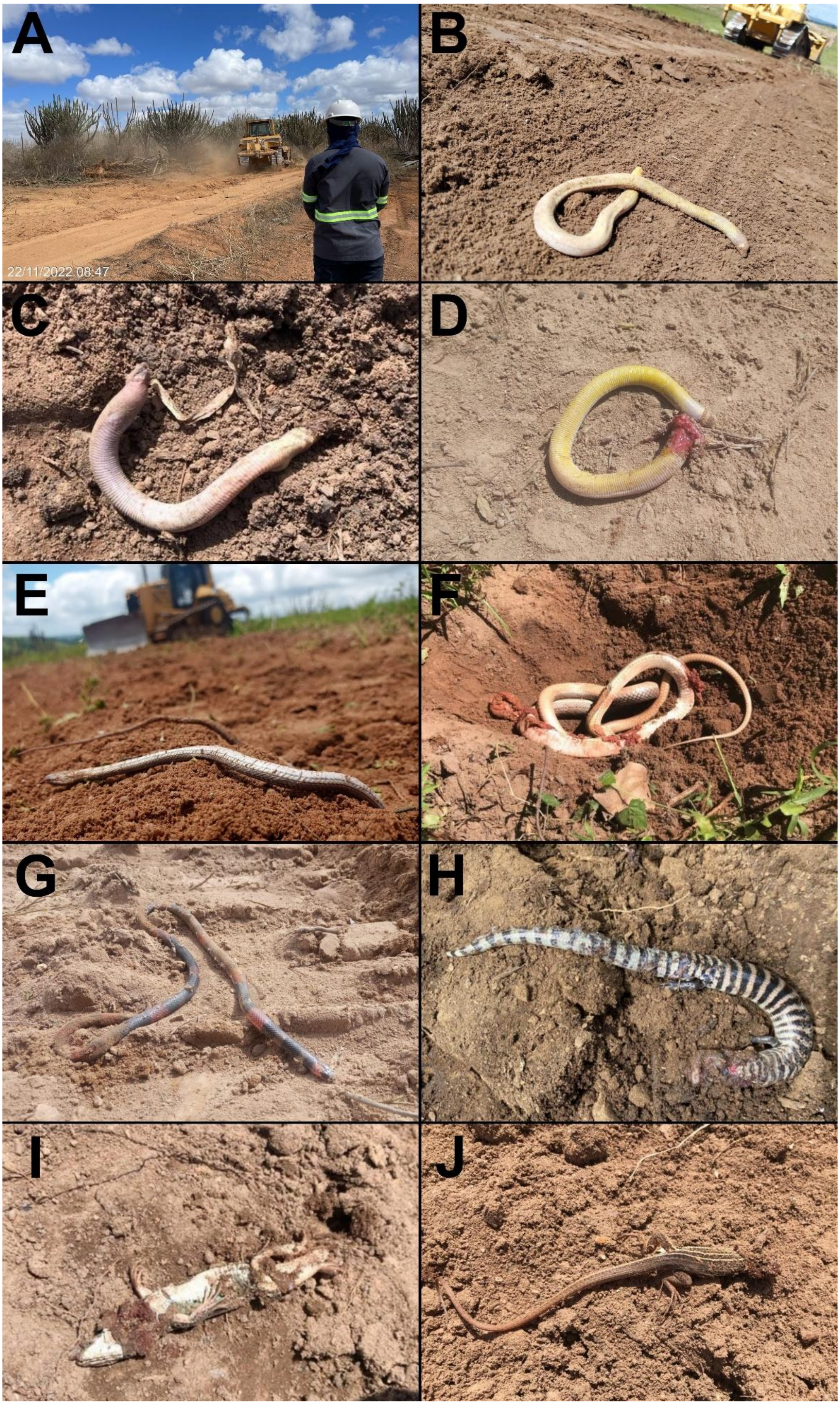

Online Resource 1. Examples of animal mortality during vegetation clearing and earthmoving stages of wind and solar energy developments. (A) Biologist accompanying construction activities; (B–D) dead amphisbeanians; (E–G) dead snakes; (H–J) dead lizards. Photos: Planoambiental environmental consultancy company.

Online Resource 2. Total number of records, number of deaths, and mortality proportions of vertebrate species recorded by faunal rescue and deterrence programs conducted during the installation of five wind and photovoltaic energy developments in Rio Grande do Norte State, northeastern Brazil. Species are ordered in descending order by mortality rates.

Species	Faunal group	Main habitat	Body form	Number of records	Number of deaths	Mortality proportion (%)
Amphisbaena heathi	Reptilia	Fossorial	Snake-like	940	850	90.4
Leposternon polystegum	Reptilia	Fossorial	Snake-like	536	430	80.2
Amphisbaena carvalhoi	Reptilia	Fossorial	Snake-like	697	523	75.0
Amphisbaena littoralis	Reptilia	Fossorial	Snake-like	28	20	71.4
Amphisbaena alba	Reptilia	Fossorial	Snake-like	19	13	68.4
Apostolepis longicaudata	Reptilia	Fossorial	Snake-like	10	6	60.0
Apostolepis cearensis	Reptilia	Fossorial	Snake-like	206	122	59.2
Amphisbaena vermicularis	Reptilia	Fossorial	Snake-like	285	161	56.5
Erythrolamprus poecilogyrus	Reptilia	Terrestrial	Snake-like	11	6	54.5
Epictia borapeliotes	Reptilia	Fossorial	Snake-like	61	32	52.5
Amphisbaena pretrei	Reptilia	Fossorial	Snake-like	27	14	51.9
Lygophis dilepis	Reptilia	Terrestrial	Snake-like	22	10	45.5
Hydropsalis parvula	Aves	Terrestrial	Winged	9	4	44.4
Oxyrhopus trigeminus	Reptilia	Terrestrial	Snake-like	408	180	44.1
Tantilla melanocephala	Reptilia	Fossorial	Snake-like	16	7	43.8
Columbina minuta	Aves	Arboreal	Winged	14	6	42.9
Erythrolamprus viridis	Reptilia	Terrestrial	Snake-like	54	22	40.7
Micrurus bonita	Reptilia	Fossorial	Snake-like	79	32	40.5
Herpailurus yagouaroundi	Mammalia	Terrestrial	Terrestrial	5	2	40.0

			quadruped			
Cerdocyon thous	Mammalia	Terrestrial	Terrestrial quadruped	40	16	40.0
Philodryas nattereri	Reptilia	Terrestrial	Snake-like	308	121	39.3
Leptodeira tarairiu	Reptilia	Arboreal	Snake-like	18	7	38.9
Boiruna sertaneja	Reptilia	Terrestrial	Snake-like	34	12	35.3
Pseudoboa nigra	Reptilia	Terrestrial	Snake-like	84	29	34.5
Leptophis dibernardoi	Reptilia	Arboreal	Snake-like	31	10	32.3
Zenaida auriculata	Aves	Arboreal	Winged	41	13	31.7
Crotalus durissus	Reptilia	Terrestrial	Snake-like	30	8	26.7
Subulo gouazoubira	Mammalia	Terrestrial	Terrestrial quadruped	8	2	25.0
Ameiva ameiva	Reptilia	Terrestrial	Terrestrial quadruped	209	52	24.9
Bothrops erythromelas	Reptilia	Terrestrial	Snake-like	280	68	24.3
Nothura maculosa	Aves	Terrestrial	Winged	17	4	23.5
Philodryas olfersii	Reptilia	Arboreal	Snake-like	34	8	23.5
Anotosaura vanzolinia	Reptilia	Fossorial	Snake-like	26	6	23.1
Oxybelis aeneus	Reptilia	Arboreal	Snake-like	54	12	22.2
Dryophylax phoenix	Reptilia	Terrestrial	Snake-like	146	30	20.5
Coccyzus melacoryphus	Aves	Arboreal	Winged	10	2	20.0
Nothura boraquira	Aves	Terrestrial	Winged	16	3	18.8
Diploglossus lessonae	Reptilia	Fossorial	Snake-like	22	4	18.2
Thrichomys laurentius	Mammalia	Terrestrial	Scansorial	53	9	17.0

			quadruped			
Conepatus semistriatus	Mammalia	Terrestrial	Terrestrial quadruped	6	1	16.7
Corythomantis greeningi	Amphibia	Arboreal	Scansorial quadruped	52	8	15.4
Callithrix jacchus	Mammalia	Arboreal	Scansorial quadruped	40	6	15.0
Acratosaura mentalis	Reptilia	Fossorial	Snake-like	36	5	13.9
Salvator merianae	Reptilia	Terrestrial	Terrestrial quadruped	221	28	12.7
Brasiliscincus heathi	Reptilia	Terrestrial	Terrestrial quadruped	370	46	12.4
Phyllopezus periosus	Reptilia	Arboreal	Scansorial quadruped	51	6	11.8
Psychosaura agmosticha	Reptilia	Terrestrial	Terrestrial quadruped	213	25	11.7
Monodelphis domestica	Mammalia	Terrestrial	Terrestrial quadruped	44	5	11.4
Dermatonotus muelleri	Amphibia	Fossorial	Burrowing quadruped	82	9	11.0
Micrablepharus maximiliani	Reptilia	Fossorial	Snake-like	124	13	10.5
Ameivula ocellifera	Reptilia	Terrestrial	Terrestrial quadruped	7249	739	10.2
Galea spixii	Mammalia	Terrestrial	Terrestrial quadruped	149	15	10.1

Epicrates assisi	Reptilia	Arboreal	Snake-like	21	2	9.5
Euphractus sexcinctus	Mammalia	Fossorial	Burrowing quadruped	225	21	9.3
Rhinella diptycha	Amphibia	Terrestrial	Terrestrial quadruped	55	5	9.1
Athene cunicularia	Aves	Terrestrial	Winged	22	2	9.1
Leptodactylus macrosternum	Amphibia	Terrestrial	Terrestrial quadruped	203	17	8.4
Polychrus acutirostris	Reptilia	Arboreal	Scansorial quadruped	476	37	7.8
Agelaioides fringillarius	Aves		Winged	13	1	7.7
Mesoclemmys tuberculata	Reptilia	Aquatic	Aquatic	13	1	7.7
Pleurodema diplolister	Amphibia	Fossorial	Burrowing quadruped	83	6	7.2
Crotophaga ani	Aves	Arboreal	Winged	14	1	7.1
Gracilinanus agilis	Mammalia	Arboreal	Scansorial quadruped	127	9	7.1
Boa constrictor	Reptilia	Arboreal	Snake-like	144	10	6.9
Gymnodactylus geckoides	Reptilia	Terrestrial	Terrestrial quadruped	408	27	6.6
Vanzosaura multiscutata	Reptilia	Fossorial	Snake-like	1314	86	6.5
Calomys expulsus	Mammalia	Terrestrial	Terrestrial quadruped	48	3	6.3
Leptodactylus fuscus	Amphibia	Terrestrial	Terrestrial	81	5	6.2

			quadruped			
Tyrannus melancholicus	Aves	Arboreal	Winged	19	1	5.3
Iguana iguana	Reptilia	Arboreal	Scansorial quadruped	293	14	4.8
Hemidactylus brasilianus	Reptilia	Terrestrial	Scansorial quadruped	44	2	4.5
Machetornis rixosa	Aves	Arboreal	Winged	23	1	4.3
Didelphis albiventris	Mammalia	Arboreal	Scansorial quadruped	55	2	3.6
Proceratophrys cristiceps	Amphibia	Fossorial	Burrowing quadruped	28	1	3.6
Columbina picui	Aves	Arboreal	Winged	112	4	3.6
Phyllopezus pollicaris	Reptilia	Arboreal	Scansorial quadruped	172	6	3.5
Scinax x-signatus	Amphibia	Arboreal	Scansorial quadruped	259	9	3.5
Leptodactylus troglodytes	Amphibia	Terrestrial	Terrestrial quadruped	29	1	3.4
Tropidurus hispidus	Reptilia	Terrestrial	Scansorial quadruped	3353	107	3.2
Pithecopus gonzagai	Amphibia	Arboreal	Scansorial quadruped	130	4	3.1
Sporophila albogularis	Aves	Arboreal	Winged	34	1	2.9
Hemidactylus agrius	Reptilia	Terrestrial	Scansorial	488	14	2.9

			quadruped			
Lygodactylus klugei	Reptilia	Arboreal	Scansorial quadruped	460	13	2.8
Tropidurus semitaeniatus	Reptilia	Terrestrial	Terrestrial quadruped	364	10	2.7
Guira guira	Aves	Arboreal	Winged	203	5	2.5
Wiedomys cerradensis	Mammalia	Arboreal	Scansorial quadruped	124	3	2.4
Pitangus sulphuratus	Aves	Arboreal	Winged	43	1	2.3
Vanellus chilensis	Aves	Aquatic	Winged	48	1	2.1
Rhinella granulosa	Amphibia	Terrestrial	Terrestrial quadruped	156	3	1.9
Cyanocorax cyanopogon	Aves	Arboreal	Winged	104	2	1.9
Rupornis magnirostris	Aves	Arboreal	Winged	52	1	1.9
Cantorchilus longirostris	Aves	Arboreal	Winged	58	1	1.7
Chrysolampis mosquitus	Aves	Arboreal	Winged	72	1	1.4
Forpus xanthopterygius	Aves	Arboreal	Winged	72	1	1.4
Paroaria dominicana	Aves	Arboreal	Winged	153	2	1.3
Eupsittula cactorum	Aves	Arboreal	Winged	177	2	1.1
Chlorostilbon lucidus	Aves	Arboreal	Winged	90	1	1.1
Dendropsophus soaresi	Amphibia	Arboreal	Scansorial quadruped	100	1	1.0
Polioptila atricapilla	Aves	Arboreal	Winged	161	1	0.6
Caracara plancus	Aves	Arboreal	Winged	427	1	0,2

Bubulcus ibis	Aves	Terrestrial	Winged	16	0	0
Camptostoma obsoletum	Aves	Arboreal	Winged	19	0	0
Cariama cristata	Aves	Terrestrial	Winged	20	0	0
Cathartes aura	Aves	Arboreal	Winged	10	0	0
Cathartes burrovianus	Aves	Arboreal	Winged	31	0	0
Coereba flaveola	Aves	Arboreal	Winged	15	0	0
Colaptes melanochloros	Aves	Arboreal	Winged	12	0	0
Columbina passerina	Aves	Arboreal	Winged	10	0	0
Columbina talpacoti	Aves	Arboreal	Winged	17	0	0
Coragyps atratus	Aves	Arboreal	Winged	66	0	0
Coryphospingus pileatus	Aves	Arboreal	Winged	333	0	0
Crypturellus parvirostris	Aves	Terrestrial	Winged	16	0	0
Cyclarhis gujanensis	Aves	Arboreal	Winged	48	0	0
Dasypus novemcinctus	Mammalia	Fossorial	Burrowing quadruped	5	0	0
Empidonomus varius	Aves	Arboreal	Winged	33	0	0
Enyalius bibronii	Reptilia	Arboreal	Scansorial quadruped	12	0	0
Eupetomena macroura	Aves	Arboreal	Winged	23	0	0
Euphonia chlorotica	Aves	Arboreal	Winged	53	0	0
Euscarthmus meloryphus	Aves	Arboreal	Winged	19	0	0
Falco sparverius	Aves	Arboreal	Winged	14	0	0
Formicivora melanogaster	Aves	Arboreal	Winged	112	0	0
Geranoaetus albicaudatus	Aves	Arboreal	Winged	31	0	0

Glaucidium brasilianum	Aves	Arboreal	Winged	12	0	0
Glossophaga soricina	Mammalia	Arboreal	Winged	6	0	0
Hemitriccus margaritaceiventer	Aves	Arboreal	Winged	104	0	0
Herpetotheres cachinnans	Aves	Arboreal	Winged	11	0	0
Heterospizias meridionalis	Aves	Arboreal	Winged	20	0	0
Icterus jamacaii	Aves	Arboreal	Winged	50	0	0
Leistes superciliaris	Aves	Arboreal	Winged	10	0	0
Lepidocolaptes angustirostris	Aves	Arboreal	Winged	22	0	0
Leptotila verreauxi	Aves	Arboreal	Winged	22	0	0
Mimus saturninus	Aves	Arboreal	Winged	140	0	0
Molothrus bonariensis	Aves	Arboreal	Winged	12	0	0
Myiarchus tyrannulus	Aves	Arboreal	Winged	68	0	0
Myiodynastes maculatus	Aves	Arboreal	Winged	15	0	0
Myrmorchilus strigilatus	Aves	Arboreal	Winged	48	0	0
Nannochordeiles pusillus	Aves	Terrestrial	Winged	28	0	0
Nystalus maculatus	Aves	Arboreal	Winged	70	0	0
Pachyramphus polychopterus	Aves	Arboreal	Winged	16	0	0
Patagioenas picazuro	Aves	Arboreal	Winged	22	0	0
Phaeomyias murina	Aves	Arboreal	Winged	17	0	0
Picumnus limae	Aves	Arboreal	Winged	72	0	0
Pseudoseisura cristata	Aves	Arboreal	Winged	42	0	0
Tachornis squamata	Aves	Arboreal	Winged	12	0	0
Taraba major	Aves	Arboreal	Winged	19	0	0
Thamnophilus capistratus	Aves	Arboreal	Winged	49	0	0

Todirostrum cinereum	Aves	Arboreal	Winged	40	0	0
Tolmomyias flaviventris	Aves	Arboreal	Winged	67	0	0
Troglodytes musculus	Aves	Arboreal	Winged	14	0	0
Turdus amaurochalinus	Aves	Arboreal	Winged	11	0	0
Turdus rufiventris	Aves	Arboreal	Winged	23	0	0
Veniliornis passerinus	Aves	Arboreal	Winged	42	0	0
Vireo chivi	Aves	Arboreal	Winged	14	0	0
Zonotrichia capensis	Aves	Arboreal	Winged	30	0	0

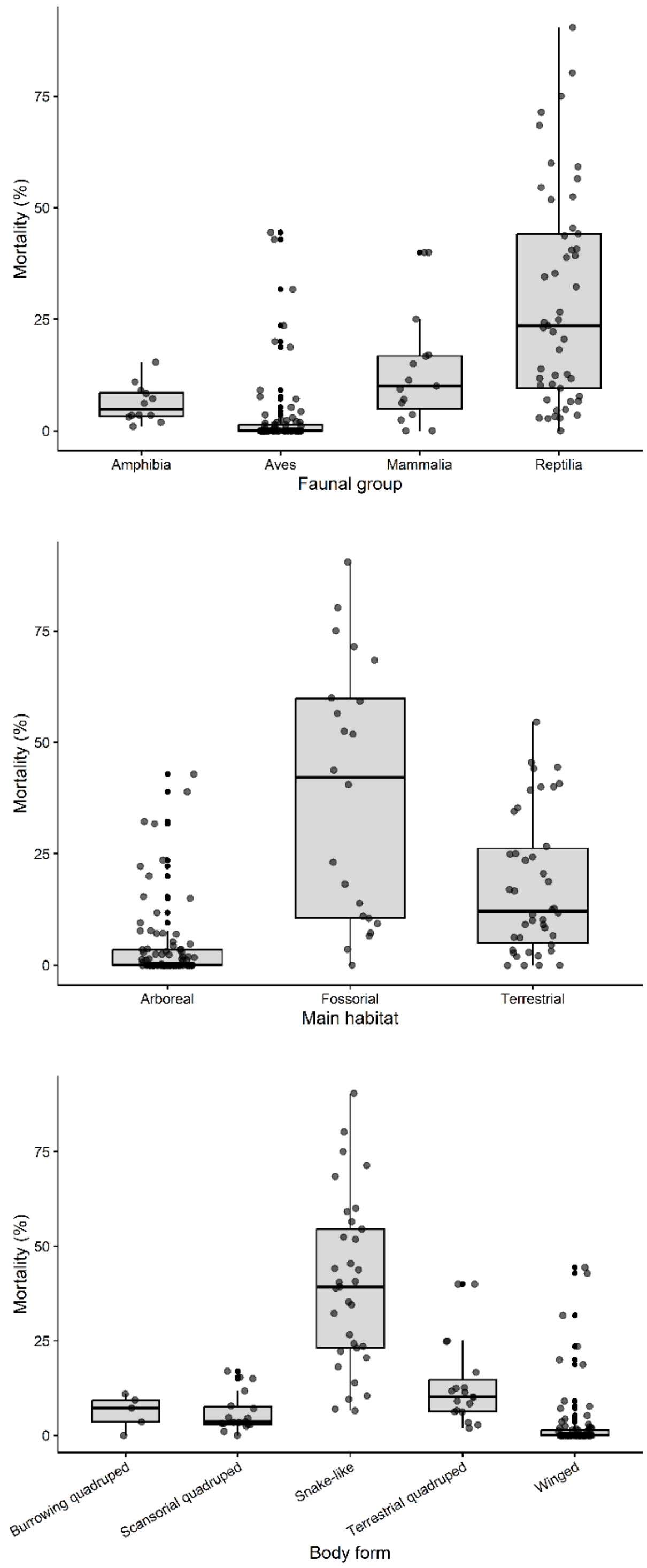

Online Resource 3. Distribution of species-level mortality proportions among (A) faunal groups, (B) habitat categories, and (C) body form categories. Boxes represent the interquartile range (25th–75th percentiles), the horizontal line indicates the median, whiskers extend to 1.5 × the interquartile range, and points represent individual species. Mortality proportion was calculated as the number of dead individuals divided by the total number of records for each species.
